# Modulation of Inflammatory Signaling Molecules in *Bordetella pertussis* Antigen-Challenged Human Monocytes in Presence of Adrenergic Agonists

**DOI:** 10.3390/vaccines10020321

**Published:** 2022-02-17

**Authors:** Md. Obayed Raihan, Brenna M. Espelien, Brett A. McGregor, Courtney Hanson, Afrina Brishti, Nathan A. Velaris, Travis D. Alvine, David S. Bradley, Matthew Nilles, Mikhail Y. Golovko, Junguk Hur, James E. Porter

**Affiliations:** Department of Biomedical Sciences, University of North Dakota School of Medicine and Health Sciences, Grand Forks, ND 58202, USA; mdobayed.raihan@und.edu (M.O.R.); brenna.espelien@ndus.edu (B.M.E.); brett.mcgregor.3@und.edu (B.A.M.); courtney.m.hanson@und.edu (C.H.); afrina.brishti@und.edu (A.B.); nathan.velaris@und.edu (N.A.V.); travis.alvine@ndus.edu (T.D.A.); david.s.bradley@und.edu (D.S.B.); matthew.nilles@und.edu (M.N.); mikhail.golovko@und.edu (M.Y.G.); junguk.hur@med.und.edu (J.H.)

**Keywords:** chemokines, prostanoid, prostaglandin, *B. pertussis*, vaccine

## Abstract

BscF is a type III secretion system (T3SS) needle protein from *Bordetella pertussis* and has previously been shown to induce a sufficient Th1 and Th17 response in human monocytes and mice as a prerequisite for long-lasting protective immunity against pertussis infection. In our current study, we aim to compare the modulation of inflammatory signaling molecules as a direct measure of the immune response to the *B. pertussis* antigens BscF and Tdap in the presence or absence of the adrenergic receptor agonists phenylephrine (PE) or isoproterenol (ISO) to observe differences that may contribute to the diminished protective immunity of the current acellular pertussis (aP) vaccine, Tdap. Stimulation of human monocyte THP-1 cells with LPS, BscF, and Tdap induced a robust elevation of CCL20, CXCL10, PGE2, and PGF2α among most chemokine and prostanoid members when compared with the control treatment. Treatment with the adrenergic agonist PE or ISO significantly enhanced the BscF- and Tdap-stimulated modulation of CCL20 and CXCL10 but not PGE2 and PGF2α, suggesting that adrenergic modulation of pertussis antigen responses might be a new therapeutic strategy to improve the longevity of pertussis immunity. Stimulation of THP-1 cells with BscF alone initiated significant expression of CXCL10 and PGF2α but not when Tdap was used, suggesting that BscF might be an important pertussis antigen for next-generation pertussis vaccines or when combined with the current aP vaccine. Our data offer opportunities for designing new therapeutic approaches against pertussis infection.

## 1. Introduction

Vaccinations, regardless of their antigenic compositions, induce immune responses to provide immune protection against specific infections [1,2]. However, vaccine-induced immune protection longevity may vary depending on the differences in antigenic compositions that initiate different immune responses against individual pathogens. The introduction of either whole-cell (wP) or acellular (aP) pertussis vaccines into the pediatric immunization schedule has reduced the morbidity and mortality related to pertussis infection to a remarkable degree. Despite the success in reducing the rate of pertussis infection with the current aP vaccine, Tdap vaccine-induced protective immunity wanes more rapidly than the previous wP vaccine, resulting in a resurgence of pertussis infection [3] in vaccinated cohorts [4]. This waning of immune protection longevity is, in part, due to insufficient immune responses elicited by Tdap vaccine antigenic compositions when compared with the previous wP vaccine that initiated strong T helper 1 (Th1) and T helper 17 (Th17) responses associated with long-lasting immune protection [5,6]. It is, therefore, important to understand the underlying cause of the insufficient immune response elicited by Tdap vaccine pertussis antigens, particularly its correlation with the generation of long-lived immune memory and the long-term persistence of these antigens to improve aP vaccine-induced immune protection longevity. The elucidation of these mechanisms will help to improve the current Tdap vaccine effectiveness as well as advance progress in designing next-generation pertussis vaccines. 

The inflammatory response is the body’s reaction to microbial invaders and is an essential part of the innate immune response [7], which plays a critical role in T cell priming and shaping the subsequent adaptive immune responses [8]. Inflammatory signaling molecules or mediators of inflammation are mostly derived from immune defense cells and plasma components [9,10], which mediate the inflammatory response [11]. Although an appropriate inflammatory response is critical for combatting microbial invaders, an excessive inflammatory response might lead to tissue damage and a variety of diseases. Tight regulation of inflammatory mediators that mediate inflammatory responses is, thus, critical in organizing long-lived adaptive immunity for long-lasting immune protection against specific pathogens. 

Chemokines are secreted inflammatory signaling proteins that belong to a family of cytokines that act as chemoattractants to guide immune cell migration [12]. Chemokines play a significant role in the initiation of the immune response, regulating the development and function of memory T cells, which are key to the adaptive immune process for providing antigen-specific stable immune protection [13,14]. Prostaglandins are a member of the prostanoid family of bioactive lipid mediators, which regulate the maturation, migration, and activation of several immune cells of both immune systems [15,16] to modulate the strength, quality, and duration of immune responses [17,18,19]. In addition, prostaglandins have a regulatory function in T cell immunity; for example, prostaglandin E2 (PGE2) aids in T-cell proliferation to potentiate the Th1 and Th17 responses [20] that are critical in providing long-lasting protective immunity. The adequate quantification of inflammatory signaling molecules or mediators of inflammation that control the immune and inflammatory response is, therefore, critical for elucidating vaccine antigen-induced protective immunity against specific pathogens. 

Adrenergic systems have been implicated in stress-mediated changes to immune responses [21]. Many cells of both innate and adaptive immune systems express adrenergic receptors (ARs) [22], and adrenoceptor activation or adrenergic signaling of the sympathetic nervous system are known to modulate antigen-initiated immune responses by regulating a variety of functions in immune cells, including cell migration and cytokine secretion [23,24]. Both the sympathetic and parasympathetic nervous systems can activate and amplify local inflammatory responses to contain or eradicate microbial invaders and restore host homeostasis [25]. In agreement with the findings of other researchers [26,27,28], our previous studies using THP-1, a human immortalized monocyte cell line that expresses β-AR, demonstrated significant modulation of the inflammatory response in the presence of AR agonists [29,30,31]. Adrenergic modulation of immune or inflammatory responses in pertussis infection offers, therefore, a potential therapeutic approach to improve vaccine antigen-induced immune protection longevity. 

Tdap vaccine components include tetanus and diphtheria toxoids together with three or more pertussis antigens, including pertussis toxin (PT), filamentous haemagglutinin (FHA), fimbrial proteins 2 and 3 (FIM 2 & 3), and pertactin (PRN) [32]. Previous studies report the immunomodulatory impact of individual pertussis antigens present in the Tdap vaccine on chemokine expression including: PT, FIM, and FHA [33,34,35]. Bacterial type III secretion needle proteins are known pathogen-associated molecular patterns (PAMPS) that induce production of pro-inflammatory cytokines through the stimulation of Tlr2 and Tlr4 signaling [36,37]. YscF, from *Yersinia pestis*, has been shown to be a protective antigen against *Y. pestis* infection [38]. Antibody isotyping analysis following immunization of mice with YscF suggested that YscF drove a strong Th1 and Th2 immune response in the presence of complete Freund’s adjuvant [38]. Furthermore, Osei-Owusu et al. demonstrated that T3SS needle proteins have a secondary function during infection of modulating host innate immune responses [36]. Preliminary studies show that immunization of mice with BscF generates a Th1 and Th17 immune response [5,6] and unpublished data. These observations with T3SS needle proteins suggest that needle proteins can help to modulate immune responses, leading to the hypothesis that needle proteins could provide adjuvant activity in vaccine formulations. Here, in our current study, we aim to compare the profile of the immune responses modulated by pertussis antigen BscF or Tdap in the presence or absence of adrenergic receptor agonists. We examined both chemokine and prostaglandin profiles as a direct measure of the immune response generated from cultured human monocytes after stimulation with Tdap, BscF, and LPS, in the presence or absence of PE or ISO, to provide new insights into the possible mechanisms of Tdap vaccine-associated diminished immune protection and suggest new therapeutic strategies for next-generation pertussis vaccine development. 

## 2. Materials and Methods

### 2.1. Chemicals

We purchased LPS from Sigma-Aldrich, St. Louis, MO, USA (Cat # L2654), trypsin from Invitrogen (Cat # 25200-072), and HEPES from Thermo Fisher scientific, Waltham, MA, USA (Cat # BP310-1). THP-1 cells (ATCC^®^TIB-202TM) were obtained from Dr. Matthew Nilles’s laboratory, University of North Dakota. THP-1 growth media (RPMI 1640: Media) was acquired from Gibco, Thermo Fisher scientific, Waltham, MA, USA (Cat # SH30537.03), penicillin/streptomycin from Gibco, Thermo Fisher scientific (Cat # 15140-122), and 6-well sterile tissue culture plates from Corning Life Sciences, Tewksbary, MA, USA (Cat # 430167). PGD2, PGE2, PGF2α, PGI2, and TXA2 were purchased from Cayman Chemical (Ann Arbor, MI, USA). Methanol and acetonitrile of HPLC grade were obtained from EMD Millipore (Billerica, MA, USA). Formic acid (reagent grade) was purchased from Sigma-Aldrich (St. Louis, MO, USA). Deionized water was purified via a Milli-Q system from EMD Millipore, and cell-culture grade water was obtained from Mediatech, Inc. (Manassas, VA, USA). 

### 2.2. Cell Culture and Treatments

Human immortalized monocytes (THP-1) originally from ATCC (Manassas, VA, USA) were trypsinized with 0.25% trypsin (Gibco, Cat#12604-021) and centrifuged at a speed of 300× *g* for 5 min. The cells were seeded in RPMI media without serum at a density of 10^6^ cells/mL and were incubated in 5% CO_2_ at 37 °C for 1 h prior to antigen stimulation and agonist treatment (e.g., with or without LPS, BscF, Tdap, and/or with or without PE or ISO). After agonist addition, cells were incubated in the same conditions for 24 more hours, after which the conditioned media were isolated and used to quantitate the amounts of chemokines or prostaglandins secreted using a fluorescent multiplexed bead-based immunoassay (FMIA) or ultra-performance liquid chromatography with tandem mass spectrometry (UPLC–MS/MS), respectively. The pertussis antigens evaluated in the study were prepared fresh immediately before administration. INFANRIX (GSK) acellular pertussis (aP) vaccine (Tdap) and purified recombinant needle protein (BscF, Nilles lab) were used in the study. Tdap was diluted with PBS to 1/12th of the human dose (based on total antigen content). Purified BscF was prepared by dissolving 50 mg in 2.5 mL sterile filtered 1 × PBS and added to a final concentration of 1.0 µg/mL. Tdap preparation was brought into solution by a 1:30 dilution with sterile filtered 1 × PBS and subsequent vortexing. 

### 2.3. Fluorescence Multiplex Bead-Based Immune Assay (FMIA)

Chemokines were analyzed in the cell supernatant from THP-1 cells using LEGENDplex (BioLegend, San Diego, CA, USA) proinflammatory chemokines and inflammation panels, following the manufacturer’s instructions. These panels allow simultaneous quantification of MCP-1, MCP-2, RANTES, IP-10, Eotaxin, Eotaxin-2, TARC, MIP-1α, MIP-1β, MIG, MIP-3α, ENA-78, GROα, I-TAC, IL-8, IL-1β, IFN-γ, TNF-α, IL-6, and IL-10. Briefly, separated conditioned media were spun at 1000× *g* for 10 min to remove the debris and were assayed immediately. Lyophilized chemokines standard cocktails were reconstituted in 250 µL assay buffer and added to propylene microcentrifuge tubes labeled as top standard C7 (10 ng/mL). Six other microcentrifuge tubes for the standard sample were labeled as C6, C 5, C4, C3, C2, and C1 and 75 µL assay buffer was added to each. A 1:4 dilution of the top standard C7 was made by transferring 25 µL of the top standard C7 to the C6 tube and mixing well. Similarly, 1:4 dilutions were made to obtain C5, C4, C3, C2, and C1 standards. Assay buffer was used as the 0 pg/mL standard (C0). The assay was performed using a V-bottom plate, and standard samples were loaded into the first two columns for better data acquisition and analysis. The standard well was loaded with 25 µL assay buffer and 25 µL standard, whereas sample wells were loaded with 25 µL assay buffer and 25 µL undiluted cell supernatant. After loading samples and standards into the plate, 25 µL reconstituted bead mix was added to each well. The plate was then placed in the centrifuge at 250× *g* for 5 min. Immediately after centrifugation, the supernatant was removed by quickly inverting and flicking the V-bottom plate. The remaining supernatant was drained by blotting the plate on a stack of clean tissue paper. Both sample and standard wells in the plate were washed by adding 200 μL of washing buffer and incubating for one minute in the dark. Following centrifugation, the supernatants from individual wells were removed. Each well was loaded with 25 µL of detection antibodies prior to sealing the plate with a plate sealer and covering with aluminum foil to protect it from light exposure. The plate was then vortexed using a shaker at 250× *g* for 1 h at room temperature. After incubation, each well was loaded with 25 µL of SA–PE (Streptavidin R-Phycoerythrin) directly, and the plate was resealed with a new plate sealer. The aluminum foil-wrapped plate was then vortexed again on a shaker at 250× *g* for 30 min at room temperature. Following incubation, the supernatant was removed as described above. The bead mix was resuspended within each well using 150 µL of 1× wash buffer. Fluorescence signals were then measured by flow cytometry.

### 2.4. Prostanoids Extraction and Quantification by UPLC–MS/MS

We measured prostanoids levels in the conditioned media using ultra-performance liquid chromatography with tandem mass spectrometry (UPLC–MS/MS) following our earlier published method [39]. First, prostanoids internal standards were extracted from the conditioned media following a single-step methanol extraction protocol, and then, the individual prostanoid level was quantified using a stable isotope dilution approach against the respective internal standard. In brief, methanol extraction was performed by mixing conditioned media into methanol at a ratio of 1 to 7.5. The sample mix was sonicated 2 times, 7 s each time with a power output of 50J (Model 150 Sonic Dismembrator, Thermo Fisher scientific, Waltham, MA, USA), vortexed for 5 min, followed by centrifugation at a speed of 10,000× *g* for 15 min at 4 °C. The supernatant was next transferred to inert microinserts and kept in a −80 °C freezer for 10 min to precipitate additional proteins. The samples were further centrifuged at 1000× *g* for 10 min at 4 °C, and the supernatant was transferred to a new microinsert if any additional protein precipitates were observed after warming the samples. Simultaneous detection of individual prostanoid concentrations was measured using a quadrupole time-of-flight mass spectrometer (Q-TOF, Synapt G2-S, Waters, Milford, MA, USA) connected to an electrospray ionization ion source. MassLynx V4.1 software was used for instrument control, acquisition, and sample analysis. The standard curves were generated using a constant concentration of isotope-labeled internal standard (100 pg on column) and variable concentrations of individual prostanoids (1 pg to 10 ng on the column). Prior to injection on the column, 90 μL of the extract was spiked with either 1 ng prostanoid diluted in 10 μL methanol or with 10 μL methanol alone to correct for endogenous prostanoid levels. One nanogram of prostanoid in 100 μL methanol was analyzed as a separate sample. Data analysis was carried out with Sciex MultiQuant 2.1.1 (AB Sciex LLC, MA, USA). 

### 2.5. Statistical Analysis

All experiments in independent experimental groups were performed in triplicate, and GraphPad Prism version 5.0f (GraphPad Software, La Jolla, CA, USA) was used to assemble data into bar graphs. An ordinary one-way analysis of variance (ANOVA) was used to determine whether there were any differences between the means of n = 3 independent experiment. A Dunnett multiple comparisons test was subsequently used to determine significance (**** *p* < 0.0001).

## 3. Results

### 3.1. THP-1 Cell Expression of Chemokine CCL20 (MIP-3α) in Response to LPS or B. pertussis Antigens, BscF and Tdap Are Modulated by Adrenergic Agonists

**Figure 1:**
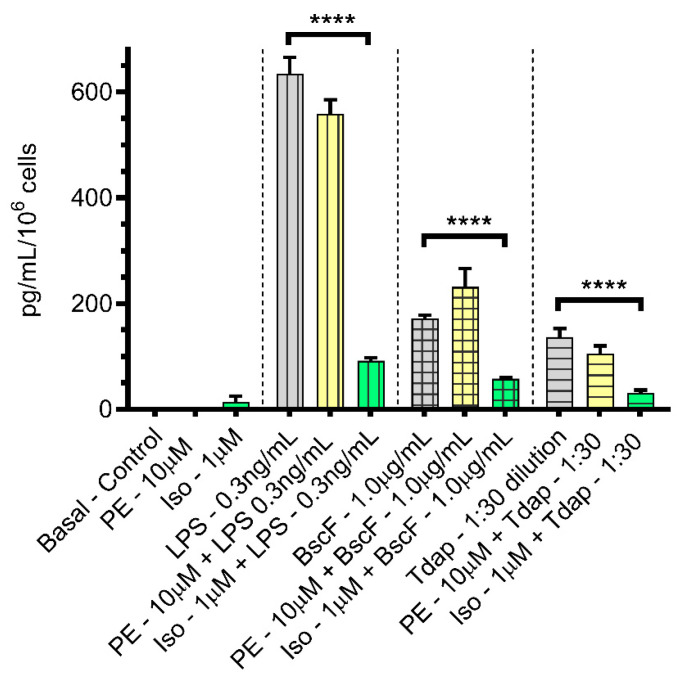
Both PE and ISO attenuated CCL20 expression, upregulated by LPS or the *B. pertussis* antigens, BscF, and Tdap in THP-1 cells. THP-1 cells in serum-deprived RPMI media treated with PBS, PE alone (10.0 µM), and ISO alone (1.0 µM) as control treatments, LPS alone (0.3 ng/mL), BscF alone (1.0 µg/mL), and Tdap alone (1:30 dilution) as antigen stimulation and LPS + PE (0.3 ng/mL + 10.0 µM)/LPS + ISO (0.3 ng/mL + 1.0 µM), BscF + PE (1.0 µg/mL + 10.0 µM)/BscF + ISO (1.0 µg/mL + 1.0 µM), and Tdap + PE (1:30 dilution +10.0 µM)/Tdap + ISO (1:30 dilution +1.0 µM) as agonist-treatment groups. After 24 h incubation, conditioned media from both control and treatment groups were collected, and chemokine levels were quantitated with FMIA. The data presented are the mean and standard error of triplicate assays. Statistically significant changes are indicated by **** *p* < 0.0001.

We measured 20 chemokines with both pro- and anti-inflammatory properties based on commercially available antibodies and found that only two chemokines—CCL20 and CXCL10—were modulated significantly compared with LPS treatment alone. Significantly increased amounts of CCL20 (Figure 1) over basal level in control treatments were secreted from THP-1 monocytes stimulated with LPS (vertical lines), BscF (checkered lines), or Tdap (horizontal lines). There was no significant modulation of the immune response when PE (yellow) was included during the incubation period. However, there was a significant reduction in the amount of CCL20 secreted in the presence of ISO when compared with antigen alone. CCL20 acts as a chemoattractant by binding to CCR6 receptors, which are expressed on memory T cells and immature dendritic cells (iDCs). Memory T cells are long-lived antigen-experienced cells that provide long-lasting protection against repeated infections with the same pathogen [40].

### 3.2. THP-1 Cell Expression of Chemokine CXCL10 (IP-10) in Response to LPS or B. pertussis Antigens, BscF and Tdap Are Modulated by Adrenergic Agonists

**Figure 2:**
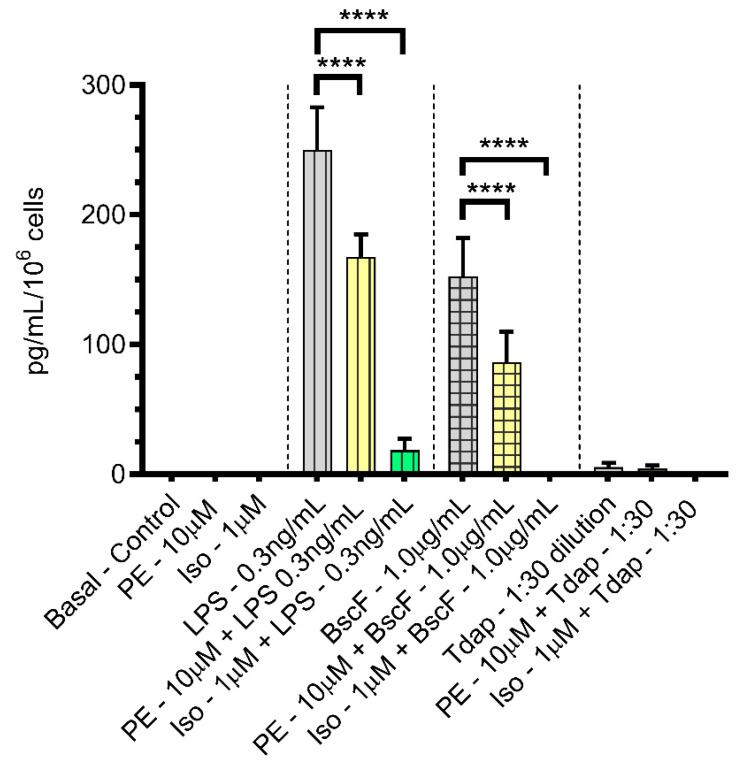
Both PE and ISO reduced the expression of chemokine CXCL10 upregulated by LPS or *B. pertussis* antigens, BscF and Tdap, in THP-1 cells. THP-1 cells treated with PBS, PE alone (10.0 µM), and ISO alone (1.0 µM) as control treatments, LPS alone (0.3 ng/mL), BscF alone (1.0 µg/mL), and Tdap alone (1:30 dilution) as antigen stimulation and LPS + PE (0.3 ng/mL + 10.0 µM)/LPS + ISO (0.3 ng/mL + 1.0 µM), BscF + PE (1.0 µg/mL + 10.0 µM)/BscF + ISO (1.0 µg/mL + 1.0 µM), and Tdap + PE (1:30 dilution +10.0 µM)/Tdap + ISO (1:30 dilution +1.0 µM) as agonist treatments. Twenty-four hours later, conditioned media from both the control and treatment groups were collected, and chemokine levels were quantitated with FMIA. Data in the bar graph presented as mean ± SEM of triplicate assays. A statistically significant change between control and treatment groups is indicated by **** *p* < 0.0001.

Inflammatory chemokine IFN-gamma-inducible protein-10 (IP-10/CXCL10) is the second chemokine we found to be modulated by adrenergic agonist treatment among all 20 chemokines tested. Significantly increased amounts of the chemokine interferon-gamma-inducible protein-10 (Figure 2) over basal amounts in the control treatment were secreted from monocytes stimulated with the antigens LPS (vertical lines) and BscF (checkered lines) but not with Tdap (horizontal lines). There was a significant reduction in the amount of CXCL10 secreted in the presence of PE (yellow) or ISO when compared with the respective antigen treatment alone. CXCL10 is primarily known as an “inflammatory” chemokine that binds to the CXCR3 receptor and mediates the transmigration of memory T cells [41]. Memory T cells interact with the CXCR3 receptor to be re-localized in lymph nodes and potentiate protective immunity against pathogens upon repeated infection with the same pathogen [42]. 

### 3.3. Immunomodulation of PGE2 Expression in THP-1 Cells in the Presence of Adrenergic Agonists

**Figure 3:**
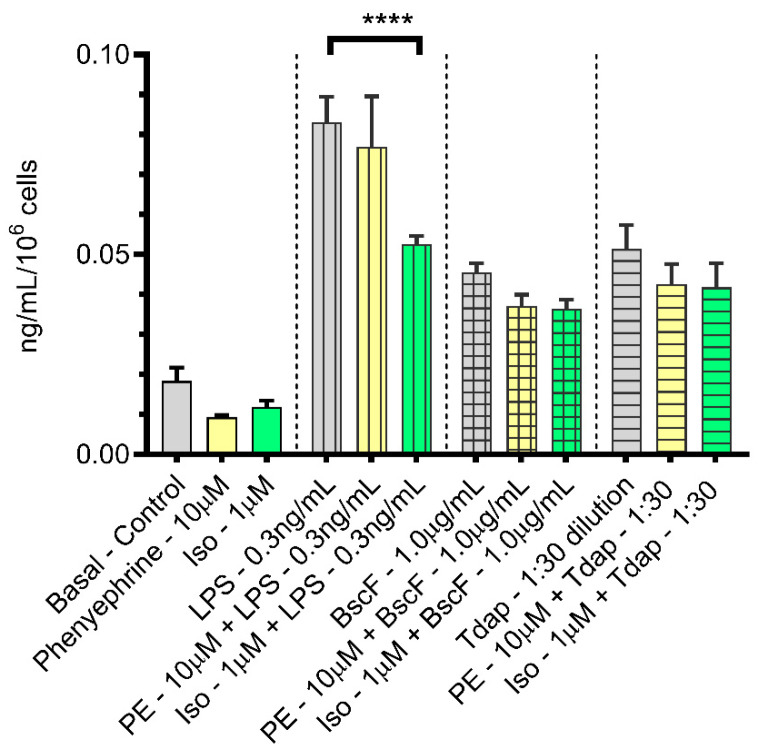
Both PE and ISO failed to significantly attenuate the expression of the prostanoid family member PGE2, upregulated by LPS or *B. pertussis* antigens, BscF and Tdap, in THP-1 cells. THP-1 cells in serum-deprived RPMI media treated with PBS, PE alone (10.0 µM), and ISO alone (1.0 µM) as control treatments, LPS alone (0.3 ng/mL), BscF alone (1.0 µg/mL), and Tdap alone (1:30 dilution) as antigen stimulation and LPS + PE (0.3 ng/mL + 10.0 µM)/LPS + ISO (0.3 ng/mL + 1.0 µM), BscF + PE (1.0 µg/mL + 10.0 µM)/BscF + ISO (1.0 µg/mL + 1.0 µM), and Tdap + PE (1:30 dilution +10.0 µM)/Tdap + ISO (1:30 dilution +1.0 µM) as agonist treatment groups. Twenty-four hours later, conditioned media from both the control, antigen stimulation, and agonist treatment groups were collected, and prostanoid levels were quantitated with UPLC–MS/MS. The difference in PGE2 level between antigen stimulation and agonist treatment groups is shown as mean ± SEM. of triplicate assays. **** *p* < 0.0001 indicates a statistically significant difference when compared with antigen stimulation alone.

Next, we quantified the expression of all five prostanoid family members—prostaglandin D2, prostaglandin E2, prostaglandin F2α, prostaglandin I2 (prostacyclin), and thromboxane A2—because prostanoids are another important mediator of inflammation. Among all five prostanoid members measured, only PGE2 and PGF2α showed significant modulation with antigen stimulation. Significantly increased amounts of PGE2 (Figure 3) over the basal amount in the control PBS treatment were secreted from monocytes stimulated with the antigens LPS (vertical lines), BscF (checkered lines), or Tdap (horizontal lines). There was no significant modulation of the response when PE (yellow) was included during the incubation period. In addition, there was only a significant decrease in the amount of PGE2 secreted in the presence of ISO when compared with LPS alone but not for any of the other antigens tested. PGE2 is one of the most abundant prostaglandins produced in the body and is known to modulate the production of chemokines [43]. Endogenous PGE2 acts on cells of both immune systems and suppresses multiple immune functions [44,45]. In bacterial infection, the inhibition of PGE2 production and signaling may represent an important therapeutic strategy to treat bacterial infections [46]. 

### 3.4. Immunomodulation of PGF2α Expression in THP-1 Cells Treated with LPS or B. pertussis Antigens in the Presence of Adrenergic Agonists

**Figure 4:**
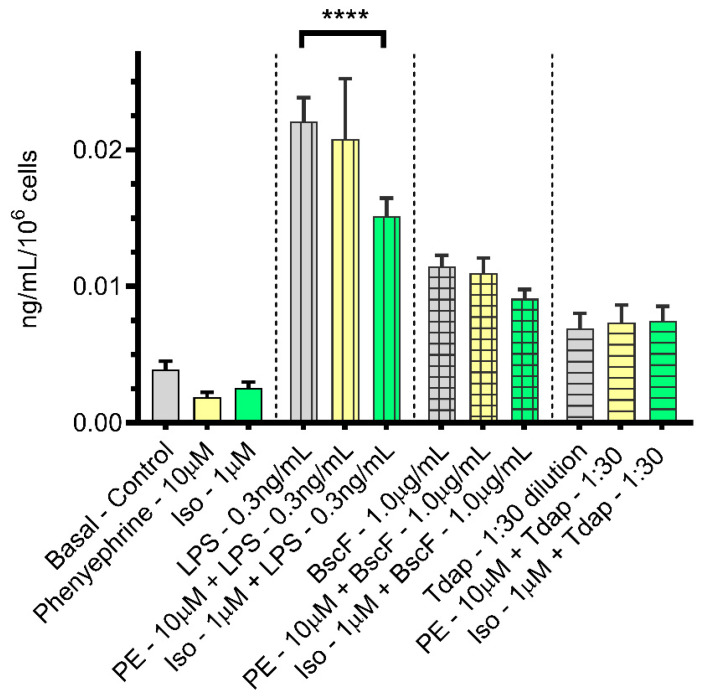
Both PE and ISO failed to significantly attenuate the expression of the prostanoids family member PGF2α upregulated by LPS or *B. pertussis* antigens, BscF and Tdap, in THP-1 cells. THP-1 cells in serum-deprived RPMI media treated with PBS, PE alone (10.0 µM), and ISO alone (1.0 µM) as control treatments, LPS alone (0.3 ng/mL), BscF alone (1.0 µg/mL), and Tdap alone (1:30 dilution) as antigen stimulation and LPS + PE (0.3 ng/mL + 10.0 µM)/LPS + ISO (0.3 ng/mL + 1.0 µM), BscF + PE (1.0 µg/mL + 10.0 µM)/BscF + ISO (1.0 µg/mL + 1.0 µM), and Tdap + PE (1:30 dilution +10.0 µM)/Tdap + ISO (1:30 dilution +1.0 µM) as agonist treatment groups. Twenty-four hours later, conditioned media from both the control, antigen stimulation, and agonist treatment groups were collected, and prostanoid levels were quantitated with a UPLC–MS/MS assay. The difference in PGF2α level between antigen stimulation and agonist treatment groups is shown in the bar graph as average with the standard error of average in triplicates. **** *p* < 0.0001 was used to indicate statistically significant values.

Significantly increased amounts of PGF2α (Figure 4) over the basal amount in the control treatment were secreted from THP-1 monocytes cells stimulated with LPS (vertical lines) and BscF (checkered lines) but not with Tdap (horizontal lines). There was no significant modulation of the response when PE (yellow) was included during the incubation period. In addition, there was only a significant decrease in the amount of PGF2α secreted in the presence of ISO when compared with LPS alone but not for any of the other antigens tested.

## 4. Discussion

Despite widespread vaccination with the current acellular pertussis (aP) vaccine Tdap, pertussis infection (whooping cough) is currently re-emerging as a major health risk among well-vaccinated populations [47,48,49], most likely due to the fading of vaccine-induced protective immunity [4]. This pertussis scenario asks for more fundamental research on vaccine immunology for a better understanding of the vaccine antigen-induced immune response, its long-term persistence, and its correlation with long-lasting protection against infection to design next-generation pertussis vaccines with a longer duration of protection. Here, in our current investigation, we used a human monocyte-like cell line—THP-1—as an in vitro cell culture model to examine the profile of inflammatory signaling molecules (chemokines and prostanoids) expression as a direct measure of the innate immune response stimulated with the pertussis antigens BscF and Tdap in the presence or absence of the adrenergic agonist PE or ISO to observe differences in immunomodulatory effects, which might contribute to the diminished Tdap vaccine immune protection longevity and may represent new therapeutic targets to improve the immune protection longevity when using the current Tdap vaccine. 

Our current study demonstrated that the adrenergic agonists modulated the immune responses (2/20 chemokines and 2/5 prostaglandins tested) generated by *B. pertussis* antigens in the human monocyte-like cell line—THP-1—in vitro. In all situations, a maximum concentration of BscF or Tdap generated a response almost equal to the response observed when using an effective concentration of LPS. Activation of the β-adrenergic receptor (β-AR) with the agonist—ISO—always modulated the chemokine response if an immune response was stimulated by a *B. pertussis* antigen. α-adrenergic receptor (α-AR) activation with the agonist—PE—also modulated the chemokine response but only for CXCL10. In all cases, AR agonists did not modulate the prostaglandin responses initiated by *B. pertussis* antigens. CXCL10 is important for the inhibition of *B. pertussis*, terminal differentiation of B-cells, and as a T-cell chemoattractant [50,51,52]. Initial loss of CXCL10 or PGF2α signaling after immunization with the acellular pertussis vaccine (Tdap) may play a role in acquired pertussis immunity. These results are the first to demonstrate a regulatory function of adrenergic agonists in the modulation of immune response when combined with the *B. pertussis* vaccine—Tdap—and further validate the role of BscF as a potential vaccine adjuvant when used in combination with adrenergic agonists. Overall, our present data suggest that the Tdap-associated fading of protective immunity may be overcome with the use of BscF as a current pertussis vaccine adjuvant and recommend adrenergic agonists as a combined therapy when using the current Tdap vaccine in pertussis infections. 

Significant advancements in our understanding of the role of the innate immune system in sensing vaccine antigens and adjuvants that activate cells of the adaptive immune system to provide immunological memory [53] have offered new immunological insights for a better understanding of the vaccine induced longevity of protective immunity. Inflammation is the immune system’s response to infection [7]. Innate immune cells’ detection of infection trigger inflammation [54] that is mediated through inflammatory signaling molecules [55]. Adaptive immunity generates immunological memory for long-lasting immune protection, and the induction of long-lived immunological memory is the basis for vaccine therapy [56]. Vaccine induced perturbations of inflammatory signaling molecules may, therefore, contribute to the modulation of the adaptive immune memory, regulating the longevity of vaccine-induced protective immunity. The inflammatory signaling molecules, such as chemokines and prostanoids, exert a regulatory function on innate immunity as proinflammatory factors [57,58] and play a critical role in shaping the resultant adaptive immune response [8]. 

Associations between stress and a higher risk of infectious illness have been reported in numerous earlier studies [26,59]. The adrenergic system plays a critical role in stress signaling [60] and the adrenergic modulation of the innate immune response and, therefore, represents novel immunomodulatory and anti-inflammatory targets for the treatment of infectious diseases [61]. Phenylephrine (PE) is a selective α-adrenergic receptor (α-AR) agonist that exerts potent anti-inflammatory effects [62] through the modulation of cytokine production, although the stimulation of α-AR is mainly associated with pro-inflammatory effects [63]. Isoproterenol (ISO) is another adrenergic receptor agonist for the β2-adrenergic receptor (β2-AR) and has been shown to induce an adaptive immune response [64]. β2-AR, which binds to stress mediators, has been reported to modulate the host response to infection by downregulating the innate immune response [26]. Our present study’s data provides evidence that either isoproterenol or phenylephrine treatment, when combined with the *B. pertussis* antigens BscF or Tdap, accelerates the modulation of inflammatory signaling molecule expression in human monocytes, consistent with earlier findings [65]. 

Interestingly, our previous study that characterized the immune-stimulating properties of BscF reported that the administration of BscF in mice and human monocytes stimulated the activation of the Th1 and Th17 response to potentiate phagocytosis and eradicated pathogens but not the Th2 response that suppresses phagocytosis [5,6], consistent with a role in providing long-term protection against pertussis by forming a long-lived immunological memory. These findings led us to examine the modulation of inflammatory mediator profiles by BscF and Tdap to understand whether the Tdap-associated fading of immune protection arises from the innate immune-regulated changes of adaptive immunity that form the immunological memory to provide long-term immune protection. 

Our present study data, in agreement with our earlier findings that characterized the immune response to immunization with T3SS system needle protein BscF from *B. pertussis* [5], demonstrate the significant immunomodulatory effect of BscF on the expression of the inflammatory signaling molecules—chemokines and prostanoids—compared with pertussis antigens in the Tdap vaccine and suggest BscF as a potential adjuvant for developing new vaccines for infants and adolescents. In addition, consistent with our earlier report [30,31], our current data suggest the adrenergic receptor agonists PE and ISO as a new therapeutic target to improve the longevity of Tdap vaccine-induced pertussis immunity because both adrenergic agonists tested accelerated the pertussis antigen-induced modulation of inflammatory signaling molecules. Our current study was designed based on the hypothesis that the fading of protective immunity conferred by an aP vaccine—Tdap—might be due to the absence of long-lived immune memory [3] that leads to an insufficient immune response in repeated infection with *B. pertussis*. However, there are data supporting evolutionary pressure change in pertussis antigens [66,67,68,69,70,71], and an evolutionary shift in the *B. pertussis* genome could be another potential cause of the waning of Tdap protective immunity that should be investigated in future studies. Additionally, our present data, generated from an in vitro human monocyte cell culture model, requires further validation in a murine *B. pertussis* respiratory model.

## 5. Conclusions

We observed significant differences in the modulation of inflammatory signaling molecules’ (chemokines and prostanoids) expression by Tdap when compared with BscF alone and in combination with adrenergic agonists in cultured human monocytes. Changes in Tdap responses in the presence of AR agonists may represent new therapeutic targets to improve the longevity of pertussis immunity while continuing the use of the acellular pertussis vaccine. Alternatively, responses using BscF only that are different from those observed with Tdap alone suggest that additional antigens might be important as an adjuvant for the current acellular vaccine. These findings may have implications for the rational design of next-generation vaccines against re-emerging pertussis infections. 

## Data Availability

The data presented in this study are available within the figures.

## Abbreviations

Tdap: tetanus-diphtheria-acellular pertussis; AR, adrenergic receptor; ISO, (−)-isoproterenol; PE, phenylephrine; aP, acellular pertussis; wP, whole-cell pertussis; LPS, lipopolysaccharide; IL-1β, interleukin-1-β; MIP-3α, Macrophage Inflammatory Protein-3 alpha; CCL20, C-C Motif Chemokine Ligand 20; CXCL10, C-X-C motif chemokine ligand 10; IP-10, interferon gamma-induced protein 10; Th1, T helper 1; Th2, T helper 2.
